# Anxiety and depression risk in Taiwan women with breast cancer and cervical cancer

**DOI:** 10.3389/fonc.2022.946029

**Published:** 2022-08-19

**Authors:** Chiu-Ming Yang, Fung-Chang Sung, Chih-Hsin Mou, Chun-Hui Liao, Po-Hui Wang, Shwn-Huey Shieh

**Affiliations:** ^1^ Department of Health Services Administration, China Medical University College of Public Health, Taichung, Taiwan; ^2^ Department of Public Health, China Medical University College of Public Health, Taichung, Taiwan; ^3^ Management Office for Health Data, China Medical University Hospital, Taichung, Taiwan; ^4^ Department of Food Nutrition and Health Biotechnology, Asia University, Taichung, Taiwan; ^5^ Department of Psychiatry, China Medical University College of Medicine, Taichung, Taiwan; ^6^ Institute of Medicine, Chung Shan Medical University, Taichung, Taiwan; ^7^ Department of Nursing, China Medical University Hospital, Taichung, Taiwan; ^8^ Department of Nursing, Asia University, Taichung, Taiwan

**Keywords:** anxiety, breast cancer, cervical cancer, depression, retrospective cohort study

## Abstract

**Background:**

Studies comparing mental disorder risks between women with breast cancer and cervical cancer are lacking. This study compared risks of developing anxiety and depression between women with breast cancer (BC cohort) and women with cervical cancer (CC cohort) using insurance claims data of Taiwan.

**Methods:**

From the 2000 to 2016 data, we identified a BC cohort and BC controls (N = 96,862) and a CC cohort and CC controls (N = 26,703), matched by propensity scores. Incident mental disorders and the Cox method estimated the related cancer cohort to control cohort hazard ratios (HRs), and 95% confidence intervals (CIs) were estimated by the end of 2016.

**Results:**

Compared to the CC cohort, the BC cohort had slightly higher incident anxiety (15.9 versus 15.5 per 1,000 person-years) and depression (6.92 vs. 6.28 per 1,000 person-years). These mental disorders were higher in respective cancer cohorts than controls. The BC cohort to BC control adjusted HRs of anxiety and depression were 1.29 (95% CI = 1.25–1.33) and 1.78 (95% CI = 1.69–1.87), respectively. The corresponding adjusted HRs for the CC cohort were 1.12 (95% CI = 1.06–1.18) and 1.29 (95% CI = 1.18–1.41). The combined incidence rates of both disorders were 1.4-fold greater in the BC cohort than in BC controls (22.8 vs. 15.8 per 1,000 person-years), and 1.2-fold greater in the CC cohort than in the CC controls (21.7 vs. 18.3 per 1,000 person-years).

**Conclusion:**

Women with breast cancer or cervical cancer are at an elevated likelihood of developing anxiety and depression disorders. These incident disorders are slightly higher in those with breast cancer.

## Introduction

Patients with anxiety and/or depression are in depressed mood and at aversion to social activity (1, 2). With more than 264 million people of all ages being affected, depression has become an important burden in medical care and public health worldwide (3, 4). These disorders can also lead to subsequent health disorders and shortened life expectancy (4). Depression and anxiety may result from biological or psychological factors with a complex interaction of socioeconomic factors. People who have health ailments or gone through adverse life events are at an elevated risk to develop anxiety and depression. In fact, depressed mood is frequently developed as a reaction to a catastrophic disorder perceived to threaten the life and wellbeing, such as in patients with cancer (5–10). Patients may suffer from cancer treatment side effects, chronic pain, self-esteem and body image, changes in quality of life and family life, and fears of recurrence, subsequent disorders, and death (11–15). Anxiety, depression, and other mood disorders are thus prevalent in cancer patients (6–8, 16–18).

Breast cancer and cervical cancer rank as the first and fourth common female cancers, respectively, with disparities in incidence and mortality worldwide (19, 20). Breast cancer is more prevalent in women in Western countries than in women in developing counties (19), whereas nearly 90% of women with cervical cancer were identified in low-income and middle-income populations (20). The breast and cervix represent the image of appearance and femininity of women. Treatments for cancer can have a negative impact on their self-image. Women with breast cancer and cervical cancer are at a high risk to be dissatisfied by changes in body image during and after the treatment procedure. Patients may also suffer from changes of sexual function and fertility, which can lead to not only sexual disfunction but also psychological adaptation (21, 22). Concurrent treatments for both cancers have been highly effective. However, survival rates have large differences internationally (23). The breast cancer survival may range from 40% in South Africa to 90% or higher in high-income countries (24). The gap of survival rates was even greater for cervical cancer, ranging from 50% to 70% (25). The 5-year survival rates also vary by the stage of the disease at diagnosis and sociodemographic status of patients. The racial disparity of survival rates was greater for cervical cancer than for breast cancer (59.8%–73.7% versus 82.2%–91.5%) in 2011–2017 in the US (26). The psychological adaptation may differ between women with breast cancer and women with cervical cancer and is associated with the disparities. Anxiety, depression, and other mood disorders developed in women with breast cancer and cervical cancer may vary by prevalence and survival among populations.

A systemic review based on seven studies found that the depression could last for years after treatment in women with CC (27). A German study interviewed a nationwide random sample of 2,141 patients with cancer and found that patients with breast cancer had the highest prevalence of mental disorder. A systematic review based on 17 studies found that the prevalence rates of anxiety ranged from 17.9% to 33.3% and of depression from 9.4% to 66.1% in breast cancer survivors (9). Studies have also associated depression with elevated cancer mortality (10).

Studies comparing the psychological adaptation between women with breast cancer and women with cervical cancer are in demand for Asian women. Cervical cancer and breast cancer ranked earlier as the first and second most common female cancers in Taiwan, with age-adjusted incidence rates of 26.27 and 20.95 per 100,000 in 1988–1993, respectively (28). Breast cancer overtook cervical cancer in 1993–1997 with incidence rates of 28.99 versus 26.82 per 100,000, respectively. The incidence gap between the two cancers increased consistently, shifting to 71.91 and 8.72 per 100,000, respectively, in 2013–2016, with cervical cancer ranking the eighth common female cancer. We suspected that the risk of developing mental disorders might be greater in women with breast cancer than women with cervical cancer, although the 5-year survival rate for breast cancer was greater than that for cervical cancer (86.8% versus 72.5%) (29). Thus, the purpose of this study was to compare the risk of developing anxiety and depression between women with breast cancer and women with cervical cancer using the insurance claims data of Taiwan.

## Methods and materials

### Data source

We used insurance claims databases and cancer registry databases from 1996 to 2016 available at the Health and Welfare Data Science Center, Ministry of Health and Welfare of Taiwan. The insurance claims data consisted of information on demographic status of insured population, longitudinal medical records of outpatient and inpatient cares, including treatments and medications provided, and costs of cares. More than 99% of residents in Taiwan have been covered in this compulsory single-payer healthcare program (30). Diseases were coded with International Classification of Diseases, Clinical Modification Ninth Revision, (ICD-9-CM), before 2016 and Tenth Revision (ICD-10-CM) since 2016. All identifications of all three data sets had been changed into the same surrogate numbers before the databases were released to users. This study was approved by the Ethical Research Committee at China Medical University and Hospital (H107257). Because personal identifications in the data files had been scrambled to protect privacy, patient consents were waived.

### Study design

**Figure 1:**
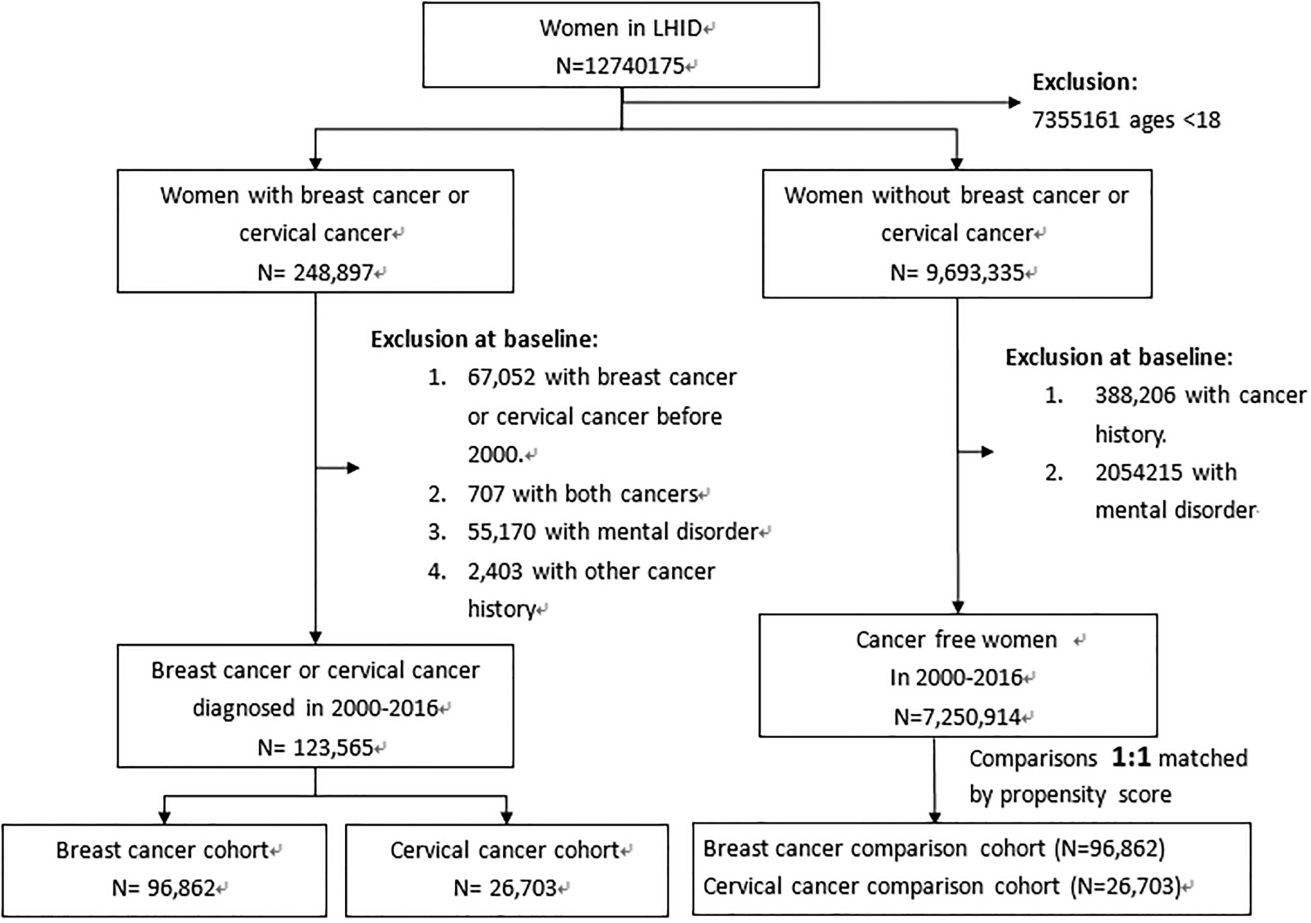
Flowchart for establishing study cohorts.

From claims data with healthcare records in the period of 2000–2016, all women aged 18 and above were identified to establish 2 pairs of cancer cohorts and control cohorts. After excluding women with cancer history and mental disorders diagnosed before 2000, we identified 96,862 women with breast cancer and 26,703 women with cervical cancer as the breast cancer cohort (BC cohort) and the cervical cancer cohort (CC cohort), respectively (**Figure 1**). The date with the cancer diagnosed was defined as the index date. Among 7,250,914 women without the history of cancer and mental disorders, we randomly selected 96,862 women as the BC cohort’s controls (BC controls) and 26,703 women as the CC cohort’s controls (CC controls), matched by the propensity score. Multivariable logistic regression estimated the propensity score for each woman with variables of age, income, urbanization level of residential areas, diagnosis year, and Charlson comorbidity index (CCI). We estimated the CCI with the sum of weighted values of comorbidities: one point was scored for myocardial infarction, congestive heart failure, peripheral vascular disease, cerebrovascular disease, dementia, chronic pulmonary disease, connective tissue disease, ulcer disease, mild liver disease, and diabetes; two points for hemiplegia, moderate or severe renal disease, diabetes with end organ damage, leukemia, and lymphoma; three points for moderate or severe liver disease; and six points for AIDS (31).

### Outcome

From the databases, we identified mental health disorders of anxiety (ICD-9: 300.0; ICD-10-CM: 110 F40 and F41) and depression: ICD-9-CM: 296.0-296.8, 300.4, and 311.X; ICD-10-CM: F32.9, F30-F33, F34.8-F34.9, and F39), which appeared in the outpatient records for at least twice or in the inpatient records for at least once. Follow-up time in person-years was calculated for each woman from the index date until the mental health disorder diagnosis, withdrawal from the insurance, death, or the end of 2016.

### Data analysis

This study used SAS Software 9.4 in Windows (SAS Institute, Cary, NC, USA) to analyze data, and 118 used P < 0.05 to indicate the significance level in comparisons. Data analysis first compared the baseline distributions, between the BC cohort and BC controls, and between the CC cohort and CC controls, including age, occupation, income, urbanization level of residential area, and CCI. The standardized mean difference of each variable between each pair of cancer cohort and control cohort was calculated to indicate the significance level. The Kaplan–Meier method was used to estimate the combined cumulative incident anxiety and depression between each pair of the cancer cohort and the control cohort. Differences were examined by the log-rank test. R software (R Foundation for Statistical Computing, Vienna, Austria) was used to plot the cumulative incidence. We calculated the incidence number and rate of each type of mental disorder for each cohort (**Table 2**). Cox proportional hazard regression analysis was used to calculate the cancer cohort to the control cohort aHR of each type of disorder. Adjustment was performed by the matched pair. The BC cohort to the CC cohort aHRs was also calculated for the two types of disorder, controlling for age, occupation, income, urbanization, and CCI score. Incidence rates of anxiety and depression (per 1,000 person-years) were then pooled as the overall rate calculated for each cohort by the baseline variables. Cox proportional hazard regression analysis was also used to calculate the cancer cohort to the control cohort adjusted hazard ratios (aHRs) and 95% confidence intervals (CI) by these variables. Adjustment was performed by the matched pair.

## Results

**Table 1** shows that distributions of all baseline variables were not different between the BC cohort and BC controls and between the CC cohort and CC controls. The cancer cohort was slightly older than their controls in both pairs (mean ages 52.3 versus 51.7 years for the BC pair and 56.6 versus 56.1 for the CC pair). Nearly 30% of women in the CC pairs and 15% of women in the BC pairs were the elderly. Compared to the BC cohort, women in the CC cohort had less white-collar jobs (18% versus 32%) with more lower incomes (52% versus 36%), living in less urbanized areas (52% versus 40%) and having a higher portion of women with a CCI score of 1 and above (14.0% versus 9.20%).

**Table 1:** Distributions of baseline characteristics compared between breast cancer cohort and BC control cohort and between cervical cancer cohort and CC control cohort.

Variable	Breast cancer N = 96,862		BC control N=96,862		Standardization difference	Cervical cancer N = 26,703		CC control N = 26,703		Standardization difference
**Age, years**	n	%	n	%		n	%	n	%	
18-39	11,988	12.4	11,964	12.4	0.001	2,915	10.9	2,908	10.9	0.001
40-49	31,919	33.0	32,199	33.2	0.006	6,555	24.6	6,592	24.7	0.003
50-64	37,843	39.1	37,906	39.1	0.001	9,270	34.7	9,151	34.3	0.009
65-74 75+	11,485 3,627	11.9 3.74	11,243 3,550	11.6 3.67	0.009 0.010	5,335 2,628	20.0 9.84	5,234 2,818	19.6 10.5	0.010 0.012
Mean (SD)	52.3	(11.9)	51.7	(13.8)	0.046	56.6	(14.4)	56.1	(16.0)	0.034
**Occupation**										
Homemaker	26,383	27.2	26,122	27.0	0.006	8,923	33.4	9,026	33.8	0.008
White collar	20,911	31.9	30,913	31.9	0.000	4,881	18.3	4,782	17.9	0.010
Blue collar	28,101	29.0	28,346	29.3	0.006	9,452	35.4	9,485	35.5	0.003
Other	11,467	11.8	11,481	11.9	0.000	3,447	12.9	3410	12.8	0.004
**Income, NTD**										
≤20,000	35,152	36.3	35,141	36.3	0.000	13,777	51.6	13,845	51.9	0.005
20,001-39,999	40,279	41.6	40,387	41.7	0.002	9,669	36.2	9,669	36.2	0.000
40,000+	21,431	22.1	21,334	22.0	0.002	3,257	12.2	3,189	11.9	0.008
**Urbanization**										
Urban	57,723	59.6	58,045	59.9	0.007	12,811	48.0	12,857	48.2	0.003
Suburban	30,659	31.7	3,791	31.8	0.003	10,118	37.9	10,215	38.3	0.007
Rural	8,480	8.75	8,026	8.29	0.017	3,774	14.1	3,631	13.6	0.015
**CCI score**										
0	87,948	90.8	89,857	92.8	0.072	22,961	86.0	23,408	87.7	0.050
1	6,053	6.25	5,106	5.27	0.042	2,281	8.54	2,086	7.81	0.027
2+	2,861	2.95	1,899	1.96	0.064	1,461	5.47	1,209	4.53	0.043

SD, standard deviation; Other: unemployed or retired; CCI, Charlson comorbidity index.

**Figure 2:**
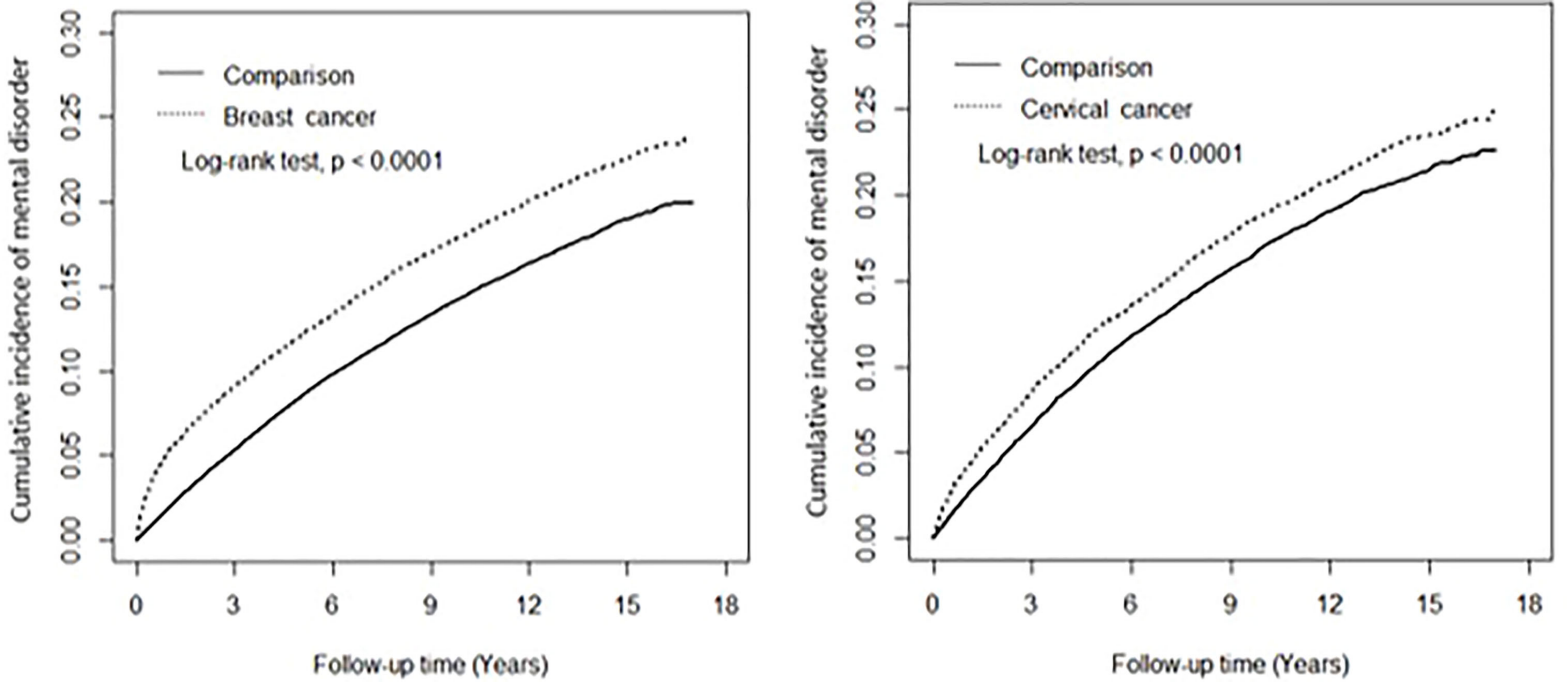
Cumulative incident anxiety and depression combined among study cohorts.

**Figure 2** shows that after the 17-year follow-up, the cumulative incidence of the two mental disorders combined in the BC cohort was approximately 3.9% higher than the BC control cohort (23.9% versus 20.0%) (log-rank test p < 0.0001), and that in the CC cohort was about 2.2% higher than in the CC control cohort (24.7% versus 22.5%) (log-rank test p < 0.0001).

**Table 2** shows that the incidence of anxiety was higher than that of depression in each cohort. The incidence of anxiety was 1.3-fold higher in the BC cohort than in the BC controls (15.9 versus 12.0 per 1,000 person-years) with an aHR of 1.29 (95% CI = 1.25–1.33). The incidence of depression was 1.8-fold higher in the BC cohort than in the BC controls (6.92 versus 3.77 per 1,000 person-years) with an aHR of 1.78 (95% CI = 1.69–1.87). The incidence rates of both anxiety and depression were slightly lower in the CC cohort than in the BC cohort, whereas these incidence rates were slightly higher in the CC controls than in the BC controls. The CC cohort to CC control aHRs for these two disorders were 1.12 (95% CI = 1.06–1.18) and 1.29 (95% CI = 1.18–1.41), respectively. Compared to the CC cohort, the incidence rates of both anxiety and depression were slightly higher in the BC cohort, but significant for depression (aHR = 1.09, 95% CI = 1.01–1.17) not for anxiety (aHR = 1.04, 95% CI 158 = 0.99–1.09) (**Table 3**).

**Table 2:** Incidence rates of anxiety and depression and cancer cohort to comparisons hazard ratio.

	Breast cancer			BC control			Hazard ratio (95% confidence interval)		
Outcome	n	PY	Rate	n	PY	Rate	Crude	Adjusted
**Anxiety**	8,724	549,703	15.9	7,617	632,488	12.0	1.33 (1.30-1.39)	1.29 (1.25-1.33)
**Depression**	3,806	549,703	6.92	2,385	632,488	3.77	1.83 (1.79-1.89)	1.78 (1.69-1.87)
	**Cervical cancer**			**CC control**				
**Anxiety**	2,584	167,182	15.5	2,754	203,189	13.6	1.14 (1.09-1.21)	1.12 (1.06-1.18)
**Depression**	1,050	167,182	6.28	960	203,189	4.72	1.33 (1.25-1. 41)	1.29 (1.18-1.41)

PY, person-years; Rate, per 1,000 person-years; Adjusted hazard ratio, adjusted for matched pair.

p < 0.001 for each hazard ratio.

**Table 3:** Breast cancer cohort to cervical cancer cohort adjusted hazard ratio of mental disorder by type.

Outcome	Crude HR (95% CI)	Adjusted hazard ratio (95% CI)
**Anxiety**	1.01 (0.94-1.03)	1.04 (0.99-1.09)
**Depression**	1.08 (1.04-1.11)	1.09 (1.02-1.17)

Adjusted for age, occupation, income, urbanization, and CCI score.

During the study period, the overall incidence of anxiety and depression was nearly 1.44-fold higher in breast cancer women than in the comparisons (22.8 versus 15.8 per 1,000 person-years, or 12,534 versus 10,005 cases), with an aHR of 1.40 (95% CI = 1.37–1.44) (**Table 4**). The incidence increased with age in both cohorts and peaked in the 65–74-year age, whereas the BC cohort to BC control cohort aHRs decreased with age, from 1.91 at 18–39 years of age to 1.15 in 65–74 years of age. The incidence decreased with income in both cohorts, whereas the HR for the BC cohort increased with income to 1.51 for those with higher incomes. Comorbidity had no contribution to the risk for the BC cohort but contributed an increased incidence for BC controls from 15.6 per 1,000 person-years in those without comorbidity to 20.7 per 1,000 person-years in those with comorbidity. The increased hazard remained significant for the BC cohort.

**Table 4:** Combined incidence of anxiety and depression and breast cancer cohort to comparison cohort adjusted hazard ratio by age, occupation, income, urbanization, and Charlson comorbidity index score.

Variable	BC control			Breast cancer			Adjusted HR (95% CI)
	Event, n	PY	Rate	Event, n	PY	Rate	
**Overall**	10,002	632,488	15.8	12,530	549,703	22.8	1.40 (1.37-1.44)***
**Age, years**							
18-39	854	95,003	8.99	1,400	78,297	17.9	1.91 (1.75-2.07)***
40-49	3,438	234,562	14.7	4,348	19,962	21.8	1.44 (1.38-1.51)***
50-64	4,182	231,962	18.0	5,039	201,127	25.0	1.36 (1.30-1.41)***
65-74 75+	983 545	44,085 26,876	22.3 20.3	1,332 411	51,600 19,069	25.8 21.6	1.15 (1.06-1.25)*** 1.07 (0.94-1.22)
**Occupation**							
Homemaker	2,862	164,289	17.4	3,480	145,502	23.9	1.34 (1.28-1.41)***
White collar	2,633	209,800	12.6	3,563	183,478	19.4	1.51 (1.43-1.58)***
Blue collar	3,370	190,807	17.7	4,147	164,392	25.4	1.40 (1.34-1.46)***
Other	1,137	67,592	16.8	1,340	57,232	23.4	1.35 (1.25-1.46)***
**Income**							
≤20,000	4,844	270,388	17.9	5,472	230,279	23.8	1.29 (1.24-1.34)***
20,001-39,999	3,409	232,941	14.6	4,639	203,872	22.7	1.51 (1.45-1.58)***
40,000+	1,749	129,159	13.5	2,419	11,553	20.9	1.51 (1.42-1.60)***
**Urbanization**							
Urban	6,008	382,364	15.7	7,462	334,402	22.3	1.39 (1.34-1.43)***
Suburban	3,119	197,532	15.8	3,859	167,820	23.0	1.41 (1.35-1.48)***
Rural	875	52,592	16.6	1,209	47,481	25.5	1.48 (1.36-1.62)***
**CCI score**							
0	9,380	602,452	15.6	11,644	510,899	22.8	1.42 (1.39-1.46)***
1+	622	30,036	20.7	886	38,804	22.8	1.12 (1.01-1.24)*

PY, person-years; Rate, per 1,000 person-years; CCI, Charlson comorbidity index.

Adjusted for matched pair. *p < 0.05, ***p < 0.001.

The overall combined incidence of anxiety and depression was near 1.2-fold higher in the CC cohort than in the comparisons (21.7 versus 18.3 per 1,000 person-years), with an aHR of 1.17 (95% CI =1.11-1.22) (**Table 5**). The incidence increased with age in both cohorts, whereas the aHR decreased with age from 1.63 (95% CI = 1.39-1.89) at 18-39 years-old to 1.07 (95% CI = 0.96-1.19) in those aged 65-74. The comorbidity associated incidence was higher in the CC cohort than in controls (24.3 versus 21.7 per 1,000 person-years), with an aHR of 1.12 (95% CI = 0.96-1.29) for CC patients, which was not significant.

**Table 5:** Combined incidence of anxiety and depression and cervical cancer cohort to comparison cohort adjusted cohort hazard ratio by age, occupation, income, urbanization, and Charlson comorbidity index score.

Variable	CC control			Cervical cancer			HR (95% CI)
	Event, n	PY	Rate	Event, n	PY	Rate	
**Overall**	3,714	203,189	18.3	3,634	167,182	21.7	1.17 (1.11-1.22)***
**Age, years**							
18-39	300	27,120	11.1	401	21,653	18.5	1.63 (1.40-1.89)***
40-49	973	59,991	16.2	952	48,342	19.7	1.18 (1.08-1.29)**
50-64	1,385	70620	19.6	1,285	58,275	22.0	1.10 (1.02-1.19)*
65-74 75+	691 365	28,134 17,324	24.6 21.1	672 324	25,667 13,245	25.6 24.5	1.07 (0.96-1.19) 1.15 (0.99-1.34)
**Occupation**							
Homemaker	1,206	65,362	18.5	1,152	52,531	21.9	1.16 (1.08-1.26)***
White collar	576	40,548	14.2	627	34,288	18.3	1.26 (1.12-1.40)***
Blue collar	1,496	73,822	20.3	1,393	62,784	22.2	1.08 (1.00-1.16)*
Other	436	23,458	18.6	462	17,579	26.3	1.37 (1.20-1.56)***
**Income, NTD**							
≤20,000	2,318	117,010	19.8	2,233	95,869	23.3	1.15 (1.09-1.22)***
20,001-39,999	1,043	63868	16.3	1,011	52,493	19.3	1.16 (1.06-1.26)**
40,000+	353	22,311	15.8	390	18,820	20.7	1.27 (1.10-1.47)***
**Urbanization**							
Urban	1,765	98,506	17.9	1,740	80,132	21.7	1.18 (1.11-1.26)***
Suburban	1,439	77,461	18.6	1,308	63,790	20.5	1.09 (1.01-1.17)*
Rural	510	27,223	18.7	586	23,260	25.2	1.32 (1.17-1.49)***
**CCI score**							
0	3,379	187,765	18.0	3,282	152,701	21.5	1.17 (1.12-1.23)***
1+	335	15,424	21.7	352	14,481	24.3	1.12 (0.96-1.29)

PY, person-years; Rate, per 1,000 person-years; CCI, Charlson comorbidity index.

Adjusted for matched pair. *p < 0.05, **p < 0.01, ***p < 0.001.

## Discussion

Mental disorder as the consequence of reaction to a catastrophic disorder may vary by type and severity of the disorder, sociodemographic variation, and stage of disorder diagnosed (8, 9, 32, 33). A population study using Japanese medical claims data evaluating mental disorders in women found that 16.9% breast cancer patients and 2.7% cervical cancer patients developed major depressive disorder (MDD) after being diagnosed with the cancers. This contrast indicates that the risk of developing the mood disorder was greater for Japanese women with breast cancer than for those with cervical cancer (33). Disorders of anxiety and depression may go hand in hand associated with the risk and severity of diseases. An anxiety disorder may trigger the occurrence of depression with a strong association with the risk level of disease. In our propensity score-matched cohort study, proportions of patients with depression developed were similar in both BC cohort and CC cohort (3.9%), and anxiety developed was slightly lower in the BC cohort than in the CC cohort (9.0 versus 9.7%). The data showed similar absolute risks of developing these mental disorders in women with BC and in women with CC, although the sample size of the BC cohort was 3.6-fold greater than that of the CC cohort, demonstrating that women in Taiwan are at a higher risk of developing BC than developing CC. The HR of developing anxiety was slightly greater for women with breast cancer than for those with cervical cancer, but not significant, featuring that both types of cancer could trigger anxiety at a similar level. The HR of developing depression was also slightly greater for women with breast cancer than with cervical cancer, but significant, featuring that BC has a slightly stronger impact than CC in triggering depression. This contrast is much slighter than that in Japanese women.

An earlier study using a smaller randomly selected database of insurance claims data of Taiwan found that women with breast cancer had significant incidence rate ratios of 1.94 for MDD and 1.22 for anxiety, relative to controls (34). Another study using a similar database found that women with cervical cancer were prominent for developing depression with an incidence rate ratio of 1.35 (35). These risk estimates are consistent with our findings. The smallish risk variations among these three studies might be associated with databases used.

Our study showed that the occurrence of both depression and anxiety in BC patients and CC patients shared similar risk characteristics associated with age: both incidence rates increased with age, but the HRs were the greatest for patients of the youngest group. The development of psychiatric disorder has been associated with comorbidities. A meta-analysis based on 40 articles showed that patients with multimorbidity could be up to three times more likely depressed than persons without chronic disorders (36). Our data failed to show this relationship in breast cancer patients, but a higher CCI was associated with slightly increased HR of combined incidence of disorders in cervical cancer patients. Comorbidities generally increase with age and are more prevalent in the elderly, explaining that the combined incidence of these two psychiatric disorders increased with age in all our cohorts. However, the subgroup analysis for HRs among age groups showed that, relative to the same age group of the respective control cohort, younger cancer patients had higher HRs than those of older age, indicating that BC or CC is the major risk factor associated with developing psychiatric disorders in younger patients, probably because comorbidities are less prevalent in younger patients than in the elderly. Therefore, the metal disorder reaction to the catastrophic diseases is stronger for younger patients.

Research has shown that social inequalities can contribute to the severity of diseases at which disadvantaged patients might be more likely to experience mood disorders (37–40). An earlier meta-analysis reported that individuals with the lowest socioeconomic position had an odds ratio of 1.81 being depressed relative to those with the highest socioeconomic position (38). The European Health Interview Survey in Spain showed that MDD in women is strongly associated with socioeconomic disadvantage, including those retired and homemakers (39). A recent meta-analysis based on 40 studies in China reported that the lifetime prevalence of MDD was the highest in participants with the lowest education or those living in rural areas (40). Our data also showed that women from disadvantaged backgrounds experienced greater incident rates of anxiety and depression, the highest for women with blue collar jobs or with a lower income in both the BC cohort and the BC controls. In the CC cohort and CC controls, CC patients of unemployed or retired or of low income were at a higher risk, and these patients are more likely older.

This study had the advantage of using a large population-based health insurance data, from which we could perform a robust cohort study design to randomly select controls matched by propensity score. The matching capacity helped minimize potential bias. The large sample sizes allowed multivariate analyses to assess the mental disorder risks associated with the sociodemographic category. There were also some limitations in this study. The claims data provided no information on the cancer stage. We therefore were unable to examine the incidence of mental disorder by the severity of cancers. Information on lifestyle was also unavailable to be included in data analysis. However, the propensity matching design and CCI use in this study could reduce potential bias, in addition to being controlled by occupation, income, and residential area. Furthermore, based on clinical diagnoses in the claims data, cancer patients with mild mental disorder symptoms might not be diagnosed. The risks of anxiety and depression may be underestimated for both cancer cohorts and comparison cohorts.

## Conclusion

Our study found that women in Taiwan are at a much higher risk of developing breast cancer than developing cervical cancer. The risks of further developing anxiety and depression are slightly higher in women with breast cancer than in women with cervical cancer, but the risk difference is significant for depression, but not for anxiety. Risks of both disorders increase with age, but relatively the hazards of developing the mental disorders were greater for youngers. Women with a less well-off economic status are also at a relatively elevated risk.

## Data availability statement

The original contributions presented in the study are included in the article/supplementary material. Further inquiries can be directed to the corresponding author.

## Ethics statement

This study was approved by the Ethical Research Committee at China Medical University and Hospital (H107257). Because Personal identifications in the data files had been scrambled to protect privacy, Patient consents were waived.

## Author contributions

Conception: C-MY, F-CS, S-SH. Design: C-MY, F-CS, C-HM, C-HL. Data analysis: C-HM. Results interpretation: C-MY, C-HM, F-CS. Data evaluation: C-HL, P-HW, S-SH. Drafting the article: C-MY, F-CS, S-SH. Manuscript revision: all authors. C-HM, F-CS, and S-SH had access to the data in the study and take responsibility for the integrity of the data and the accuracy of the data analysis. All authors contributed to the article and approved the submitted version.

## Funding

This study is supported in part by Taiwan Ministry of Health and Welfare Clinical Trial Center (MOHW110-TDU-B-212-124004), China Medical University Hospital (DMR-111-228) and China Medical University (CMU110-S-18), Taiwan. The funders had no role in the study design, data collection and analysis, the decision to publish, or preparation of the manuscript.

## Acknowledgments

We are grateful to the Health Data Science Center and China Medical University Hospital for providing administrative, technical and funding support for using the data.

## Conflict of interest

The authors declare that the research was conducted in the absence of any commercial or financial relationships that could be construed as a potential conflict of interest.

## Publisher’s note

All claims expressed in this article are solely those of the authors and do not necessarily represent those of their affiliated organizations, or those of the publisher, the editors and the reviewers. Any product that may be evaluated in this article, or claim that may be made by its manufacturer, is not guaranteed or endorsed by the publisher.
